# Partner Choice in Raven (*Corvus corax*) Cooperation

**DOI:** 10.1371/journal.pone.0156962

**Published:** 2016-06-10

**Authors:** Kenji Asakawa-Haas, Martina Schiestl, Thomas Bugnyar, Jorg J. M. Massen

**Affiliations:** 1 Department of Cognitive Biology, University of Vienna, Vienna, Austria; 2 Haidlhof Research Station, University of Vienna and University of Veterinary Medicine Vienna, Bad Vöslau, Austria; Universidad Carlos III de Madrid, SPAIN

## Abstract

Although social animals frequently make decisions about when or with whom to cooperate, little is known about the underlying mechanisms of partner choice. Most previous studies compared different dyads’ performances, though did not allow an actual choice among partners. We tested eleven ravens, *Corvus corax*, in triads, giving them first the choice to cooperate with either a highly familiar or a rather unfamiliar partner and, second, with either a friend or a non-friend using a cooperative string-pulling task. In either test, the ravens had a second choice and could cooperate with the other partner, given that this one had not pulled the string in the meantime. We show that during the experiments, these partner ravens indeed learn to wait and inhibit pulling, respectively. Moreover, the results of these two experiments show that ravens’ preferences for a specific cooperation partner are not based on familiarity. In contrast, the ravens did show a preference based on relationship quality, as they did choose to cooperate significantly more with friends than with non-friends and they were also more proficient when cooperating with a friend. In order to further identify the proximate mechanism of this preference, we designed an open-choice experiment for the whole group where all birds were free to cooperate on two separate apparatuses. This set-up allowed us to distinguish between preferences for close proximity and preferences to cooperate. The results revealed that friends preferred staying close to each other, but did not necessarily cooperate with one another, suggesting that tolerance of proximity and not relationship quality as a whole may be the driving force behind partner choice in raven cooperation. Consequently, we stress the importance of experiments that allow such titrations and, suggest that these results have important implications for the interpretations of cooperation studies that did not include open partner choice.

## 1. Introduction

Theoretical models emphasize that cooperation [1] is always at risk of exploitation [2]. In order to avoid exploitation, humans use calculated strategies such as “win-stay, lose-shift” and “generous tit-for-tat” [3]. Notably, humans prefer to cooperate with friends over strangers in iterated prisoner´s dilemma games [4], and this preference may reflect an evolved partner choice mechanism against cheating [5] which may be based on attitudes and/or emotions [6, 7].

Social animals frequently face similar types of decision-making [8], and therefore, should have evolved mechanisms that allow them to choose when it is beneficial to cooperate and when not to [9], along with mechanisms that enable them to select the most efficient cooperation partner (e.g. [10,11]). Ravens, for example, refuse to cooperate when their partner cheated in the previous trial [12], and similarly, chimpanzees adapt their level of trust in a reciprocity game based on previous trials with that individual [13], suggesting choices based on attitudes (cf. “attitudinal partner choice” [14]).

A preference to cooperate with familiar over unfamiliar individuals can be beneficial since repeated interactions, with one and the same individual, make it possible to be compensated for potential losses later (cf. reciprocal altruism [15]) and preferences to associate with familiar individuals are well known, for example in fish [16–18]. Furthermore, in several tested species the cooperation success of dyads depends on social tolerance between partners [12, 19–23]. Social tolerance is defined as tolerating another individual in close proximity (e.g. within arm’s / wing’s-length [24]), and is sometimes, but not necessarily extended to tolerance for proximity in a feeding context (e.g. co-feeding or food-sharing)[12, 19–22]. Tolerance of proximity thereby can be seen as a component of relationship quality and is therefore a prerequisite for friendship [24]. Nevertheless, these previous tests were mostly conducted in a pair-wise set-up and do not show actual partner choice. However, a recent study on captive chimpanzees, *Pan troglodytes* [25], which allowed for open partner choice at a cooperative pulling task, also revealed that social tolerance, resulting from either kinship or low rank distance positively affected mutual cooperation. Similarly, a study on wild Barbary macaques, *Macaca sylvanus* [26], which also allowed a free choice of cooperation partners, found that a strong social bond (i.e. high relationship quality) was necessary to maintain cooperation over a long period. These findings suggest that social tolerance and the quality of social bonds are both important factors for cooperation, hinting towards a long-term emotionally mediated mechanism [6,7,27]. But, not all cases of cooperation in the wild require close proximity of the collaborators. For example, cooperative hunters may need to coordinate their actions over a distance [28–31], leading to the question if social tolerance is the only factor contributing to successful cooperation and if successful cooperation necessarily relates to relationship quality.

Thus far, the primary, proximate drivers for cooperation have remained obscured, since with most of the set-ups used so far it is impossible to distinguish if partner choice in mutual cooperation settings is a *byproduct* of spatial closeness due to a high tolerance of close proximity or results from an *actual partner choice* based on relationship quality as a whole.

Ravens frequently cooperate in the wild (e.g. [32]), may even hunt cooperatively [28], and a recent study on the same captive population as this study showed that tolerance is a good predictor for ravens´ cooperation success in experimental settings [12]. Ravens indeed show differential relationships with conspecifics, whose qualities resemble those of primates [33], and they recognize and remember these relationships over several years [34]. Moreover, they understand the social relationships of others [35], and use this information to break up the newly formed cooperative alliances of others [36].

For this study, we tested eleven ravens in a set-up that allowed an individual the choice between different partners to cooperate with, by the use of two distinct loose-string paradigm apparatuses [37]. In two triadic experiments, we gave them, first, the choice between a familiar group member and a relatively unfamiliar individual—to test for a preference with respect to the degree of familiarity—and, second, between a friend and a non-friend—to test for a preference with regard to relationship quality. We further aimed to identify the proximate mechanism that causes the ravens´ preference to cooperate with specific partners; i.e., if partner choice is a result of tolerance for proximity or if partner choice is based on relationship quality as a whole. Therefore, we allowed the whole group free access to the cooperation apparatuses in a third experiment, yet in a setting that allowed us to distinguish these two mechanisms from one another.

## 2. Material & Methods

### (a) Subjects and housing

We tested seven male (♂) and four female (♀) ravens (3–5 years of age) housed in an outdoor aviary (15 x 15 x 5 m) at the Haidlhof Research Station, a joint facility of the University of Vienna and the University of Veterinary Medicine Vienna, in Bad Vöslau, Upper-Austria, AT. All subjects were born in captivity. Nine subjects (6 ♂ & 3 ♀) were hand-raised at the facility until fledging and kept together ever since, whereas two subjects (1 ♂ & 1 ♀) joined the group only 2–3 weeks before the start of the first experiment. The latter two ravens came from private persons where they were also hand-raised (see Table 1 for an overview of the subjects and their origin). Subjects were never food deprived and were being fed an appropriate and varying diet. Water was available ad libitum.

**Table 1:** Test subjects, their age, rank, sex and origin.

Name	Age (yrs)	Rank	Sex	Origin
**Laggie**	3	1	M	Bayerwald Tierpark (DE)
**Tom**	3	2	M	Bayerwald Tierpark (DE)
**Horst**	3	3	M	Spanga Gymnasium, Stockholm (SE)
**Paul**	3	4	M	Tiergarten Wels (AT)
**George**	3	5	M	Spanga Gymnasium, Stockholm (SE)
**Rufus**	3	6	M	Tierpark Stadt Haag (AT)
**Nobel**	3	7	F	Spanga Gymnasium, Stockholm (SE)
**Rocky**	4	8	M	Private owner (AT)
**Louise**	3	9	F	Spanga Gymnasium, Stockholm (SE)
**Joey**	5	10	F	Private owner (AT)
**Adele**	3	11	F	Bayerwald Tierpark (DE)

### (b) Ethics statement

This study strictly followed the statutes of the University of Vienna and was approved by the ethical board of the behavioral research group at the faculty of Life sciences, University of Vienna, (Nr: 2015–003). After the study the birds remained in their home enclosure at the Haidlhof Research Station. The individuals (i.e., experimenters) in S1 Video have given written informed consent (as outlined in PLOS consent form) to publish these case details.

### (c) Experimental design

**Fig 1:**
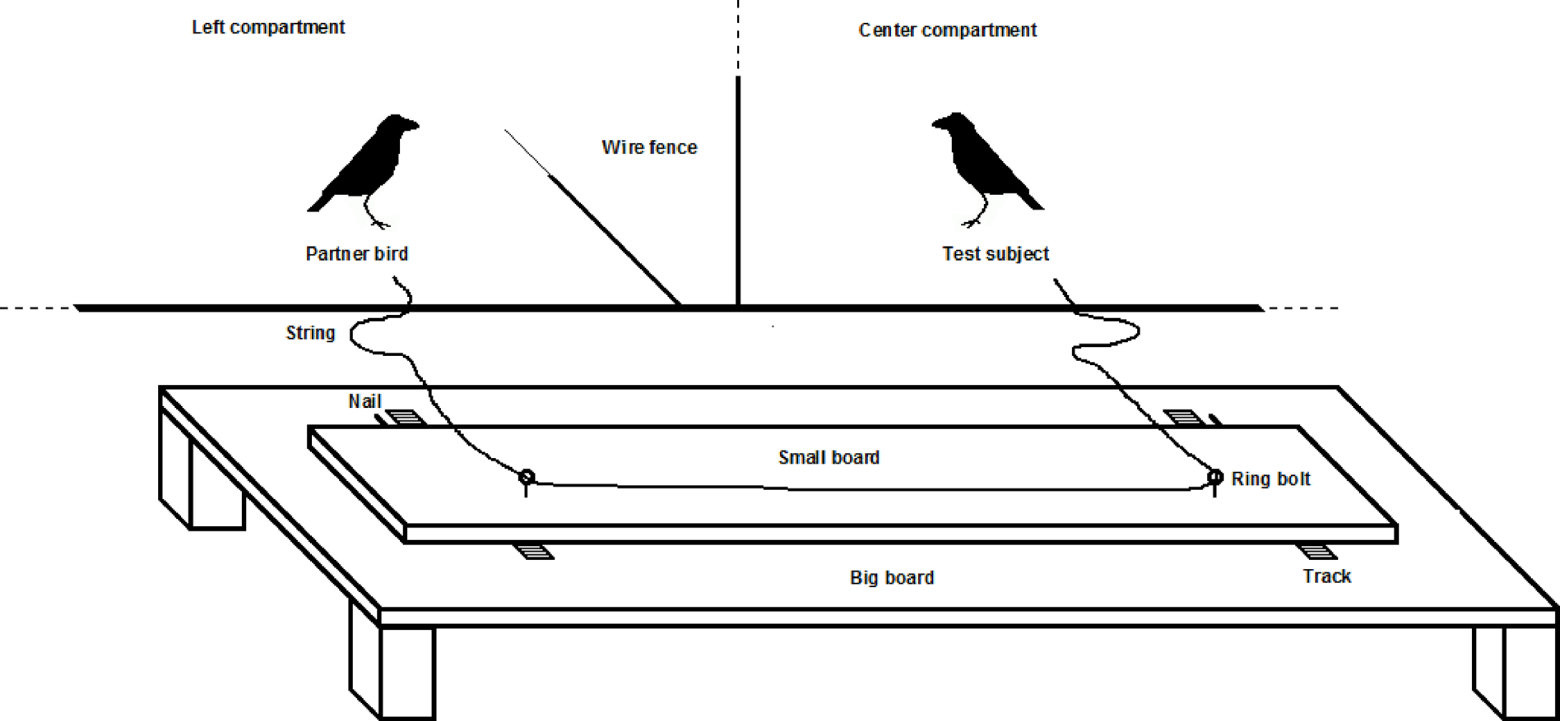
Sketch of a loose string paradigm apparatus. The small board could move along the tracks if the strings were simultaneously pulled. If only one subject pulled or if both subjects pulled asynchronously, the string was pulled through the ringbolts without affecting the small board.

The test area consisted of three adjacent compartments within the birds’ home-aviary, which were separated by a wire fence. Two loose string paradigm apparatuses (Fig 1), which required cooperation [37], were placed on the outside. Two collaborating subjects could pull food rewards (pieces of cheese of approximately 5 mm x 5 mm X 5mm placed on top of the nails; Fig 1) into reach if both birds pulled simultaneously, each at one end of the string at an apparatus. If, however, only one bird would pull the string, the string would become unthreaded and subsequent cooperation would become impossible.

#### Habituation

**Fig 2:**
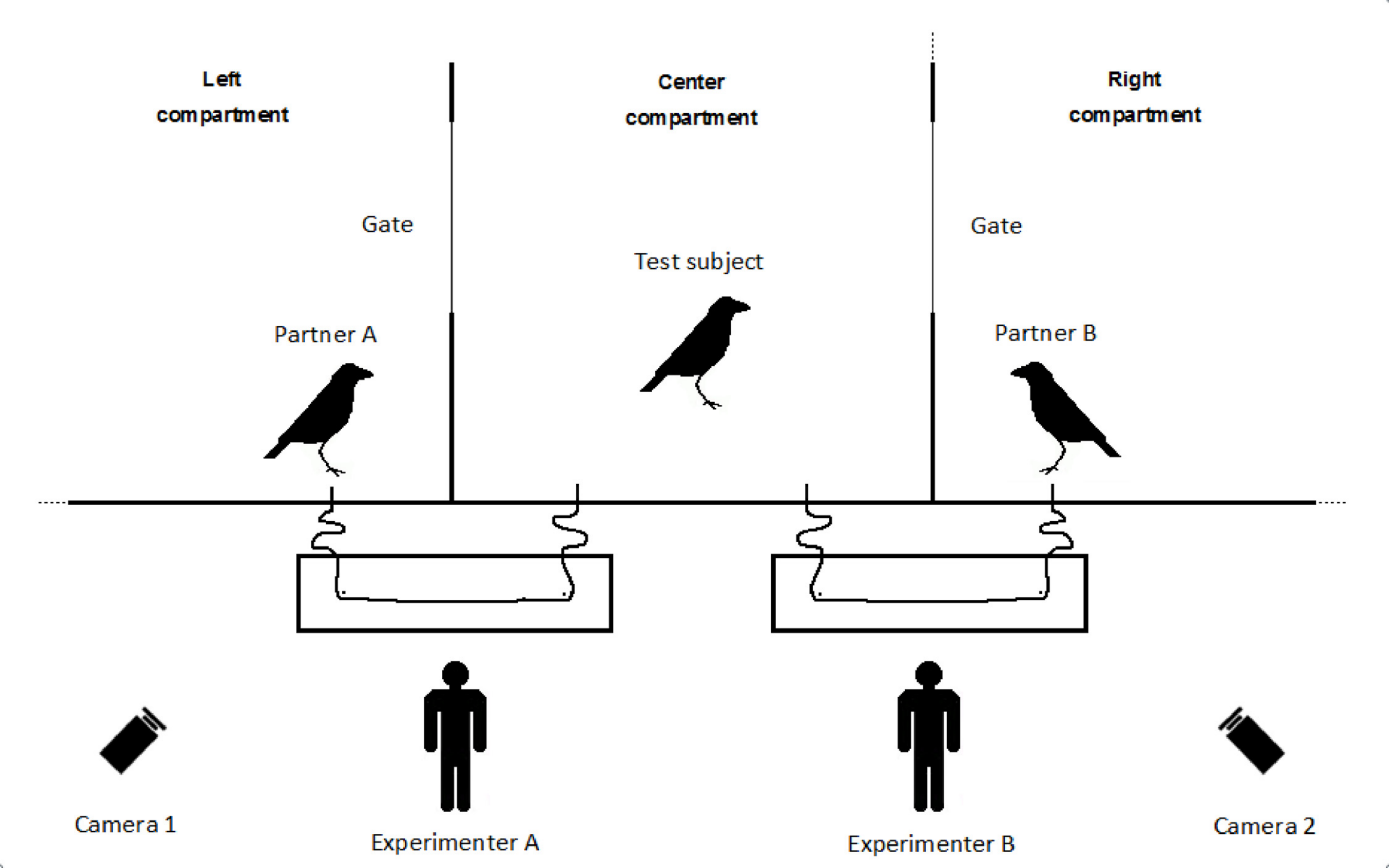
Setting of experiment No. 1 & 2. The test subject could choose to cooperate with one of the partner birds (exp. 1 = familiar vs. relatively unfamiliar, exp. 2 = friend vs. non-friend) at the beginning of every trial.

The initial 9 subjects of the group had extensive experience with the loose string paradigm, albeit with only a single apparatus, since they were tested in a previous cooperation study [12]. In contrast, the two newcomers had no experience with the paradigm. Therefore, prior to the experiments all birds were habituated to the new set-up in pairs, and had to cooperate successfully in 40 trials. These habituation trials were divided in sessions of 10 trials and over these sessions the positions of the two birds were changed such that each of the two birds had experience cooperating in each compartment (i.e. left-, middle-, and right-compartment, Fig 2). All birds needed only 4 sessions to reach the 40 successful trails criterion and thus were successful in every trial.

#### Experiment 1 & 2

In experiment No. 1 & 2, the center compartment contained the test subject, while two partner birds were placed into the adjacent areas (Fig 2). In experiment No. 1, each of the nine original group members could choose to cooperate with either a highly familiar bird (another original group member) or a relatively unfamiliar bird (one of the two newcomers) in one session of 20 trials each. Per trial the subject could, in principle cooperate twice; i.e. if both partner birds waited long enough with pulling the string for the subject to cooperate with them. A trial was ended after 2 minutes, or whenever there were no possibilities to cooperate left. A new trial was started as soon as both experimenters finished rethreading and rebaiting the apparatuses.

In experiment No. 2, all eleven birds were used as test subjects and could choose to cooperate with either a friend or a non-friend in one session of 20 trials each. Again a trial was ended after 2 minutes, or whenever there were no possibilities to cooperate left, and a new trial was started as soon as both experimenters finished rethreading and rebaiting the apparatuses. In experiment 2 we also included the two newcomers into the sample as by the start of experiment 2 they had more time (5–6 weeks in the whole) to be integrated into the group, and already showed marked preferences for particular individuals while clearly avoiding others.

We made sure that subject-partner combinations were different in both experiments to avoid reciprocation; i.e., in experiment 2 all triads were novel, as well as all dyads within those triads. Thus, each bird had 4 different partners over experiment 1 & 2 and was never the partner itself for one of those four birds.

Moreover, during test sessions of both experiments we changed the position (left or right) of the partner birds after 10 trials using positive reinforcement; i.e., we called them in / out compartments with little pieces of dog food (frolic ®). Additionally, the positions of the two experimenters, refilling the rewards and placing the ends of the strings into the compartments at the beginning of each trial, changed every 5 trials and were counterbalanced over sessions/experiments.

Experiments 1 & 2 were conducted between August 8^th^ 2014 and September 12^th^ 2014 in one succession, and depending on weather and weekends there was a maximum of 5 days in between testing days / test sessions. Per day we ran a maximum of 2 test session, and birds were in principle tested only once per day, with one exception; i.e., Rocky was used as an unfamiliar partner twice on one testing day.

In both experiments, per trial we scored which bird the test subjects chose first as cooperation partner and which bird, if applicable, as second cooperation partner. A choice was defined as whenever the bird approached a given apparatus and it became within 20 cm of it. Additionally, we scored whether after a choice, cooperation with that partner bird at that apparatus was successful or not.

#### Experiment 3

**Fig 3:**
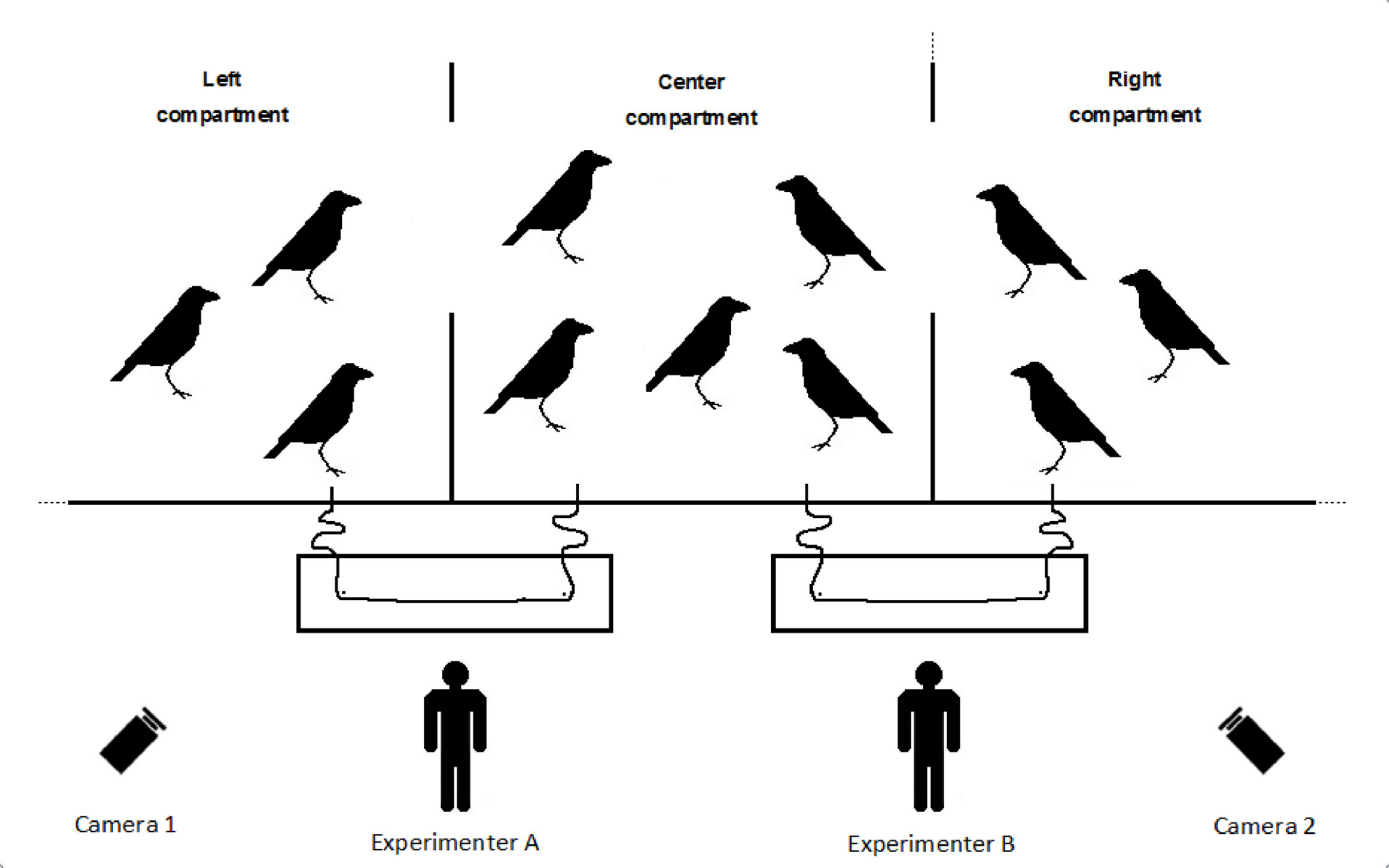
Setting of experiment No. 3. The subjects could freely move around in the whole aviary. We tested if friends stay close to each other in the center compartment while pulling with another bird (tolerance of proximity) or if friends actually cooperate with each other at one of the string pulling apparatuses (actual partner choice based on relationship quality as a whole).

In Experiment 3, the gates between the three compartments were open and all birds could and did move freely between the three test compartments and the two apparatuses (Fig 3). In this experiment the birds were tested during 7 sessions, each consisting of 100 trials. We chose for an increased number of trials to avoid monopolization of the apparatuses by the most dominant birds during all trials, since we expected these to either become satiated after a given number of trials, or go and cache their rewards somewhere else. Consequently, with this increased number of trials also low-ranking individuals would get the opportunity to participate.

Experiment 3 was conducted between October 21st 2014 and November 25^th^ 2014, we ran only one test session per day and there was a maximum of 11 days between 2 test sessions.

We scored who cooperated with whom at which apparatus (testing if friends choose to cooperate with each other and thus apply actual partner choice based on relationship quality as a whole) and who was sitting next to each other in the center compartment while pulling with another bird (testing if friends prefer tolerance of proximity over cooperation with each other). Note that although the doors separating the compartments were open, these were located at the back, therefore two individuals cooperating at one and the same apparatus would still be separated by a wire fence. Tolerance for close proximity would thus not be necessary to cooperate. In contrast, for two individuals to sit next to each other in the center compartment while pulling with another bird tolerance of close proximity was essential, though these individuals did not cooperate with each other. The positions of the experimenters were changed every 10 trials and were again counterbalanced over sessions/experiments.

### (d) Calculating social relationships

Relationship data were assessed with focal animal samplings. Focal observations of all ravens are exactly 5 min/animal, and are conducted approximately 2-3/week at our lab. We used data from April, May, August & September 2014, totaling 36.75 hours of observations (M = 3.33 ± S.E. 0.25 hours/bird). Relationship quality was measured as the rate of contact-sitting and preening of each individual with each other individual per minute (# / total focal observation minutes of both individuals in a dyad). Bouts of both behaviors are defined as not being interrupted by > 3s. Frequencies of contact-sitting and preening (cf. grooming) are regularly used as measures for relationship quality [24, 38–40]. We chose to lump these behaviors, as it is difficult to weigh their relative importance against each other. Contact sitting is bidirectional and has the benefit that it can be frequently observed among ravens, and whereas preening is rarer, it is mostly unidirectional and thereby provides us with a relative measure of importance of the relationship for each bird in the dyad.

In addition to relationship quality data, we also assessed dominance relationships between the animals in our group. Dominance ranks and hierarchy were calculated using unidirectional displacement behavior recorded from the focal samples in MatMan 1.1 [41], and the resulting fitted hierarchy was significantly linear (h’ = 0.581, n = 11, p<0.01).

To assign friends and non-friends in experiment 2, we ordered social partners from most affiliative (1) to least affiliative (10) per bird using the summed contact sitting and preening data of the focal observations obtained thus far (i.e. April, May and half of August)(cf. [42]), and chose a friend and a non-friend at the respective most extremes of this order, while also ensuring the novelty of all separate triads to avoid reciprocation within experiments No. 1 & 2. If there was a lack of affiliative data, we also used agonistic behavior yet in a conversed way. Moreover, we choose friends and non-friends that were similar with regard to rank distance to the subject. Mean rank difference between friends and non-friends was only 2.9 ± S.E. 0.59, and overall there was no significant difference between the rank distances of subject birds with either their friend or their non-friends (Wilcoxon: T+ = 30.5, N = 11, p = 0.823).

### (e) Analysis

All experiments were video recorded using two HD cameras (Canon LEGRIA HF G10). Focal animal samples were recorded with one such camera. Data for experiment 1 & 2 were coded by KA-H from video-recordings using Solomon coder (Péter, 2011; http://solomoncoder.com/). JJMM recoded 25% of those videos and inter-observer reliability was excellent (Cohen’s kappa: 1.0). Choices in the third experiment were coded live on paper with pencil by both experimenters. KA-H recoded 15% of all trials from the video-recordings and again inter-observer reliability between live coding by both the experimenters and video-coding by KA-H was excellent (Cohen’s kappa: 1.0). Finally, focal data was coded from the video-recordings by a research assistant, and the coding of focal observations by this research assistant is regularly compared to that of our main coding research assistant; their inter-observer reliability is almost excellent (Cohen’s kappa: 0.84).

We compared the number of subjects’ first choices (out of 20) and the number of subsequent successful cooperation trials with respect to the degree of familiarity (exp. 1) or relationship quality (exp. 2), with a random distribution using Wilcoxon signed-rank tests. Moreover, per subject we compared the success-rates (i.e. # successful cooperation trials / total # of opportunities) with familiar partners to those with unfamiliar partners, and with friends to those with non-friends using Wilcoxon signed-rank tests. To investigate if there was a learning effect on waiting behavior, we ran two separate binomial generalized linear mixed models (GLMM) with a logit link function on a) whether a partner waited; i.e. did it pull immediately (yes or no), and on b) whether it was successful when waiting. We included trial number per session, and the total trial numbers this individual already had as partner as fixed factors, and partner identity and actor identity as random factors. We also ran reduced models and compared models based on the comparison of corrected Akaike Information Criteria cAIC where we considered models with a smaller cAIC (the difference being >2) as the best fitting models.

For experiment 3, we ran partial row-wise matrix correlations [43] with 10000 permutations, correlating a relationship quality matrix with either a matrix of the number of successful cooperation pulls between individuals, or a matrix with the number of times individuals pulled next to each other in the center compartment, but with another bird, while partialling out the effect of rank distance. We choose to analyze our data with row-wise matrix correlations, since these control for the dependency of our data. Moreover, row-wise matrix correlations take into account the relative frequency of interactions with each individual *per* individual (rather than correlating overall frequencies), thereby avoiding overrepresentation of data of birds that participated much more (e.g. dominant birds), and are particularly robust for zero’s [41,43].

## 3. Results

**Fig 4:**
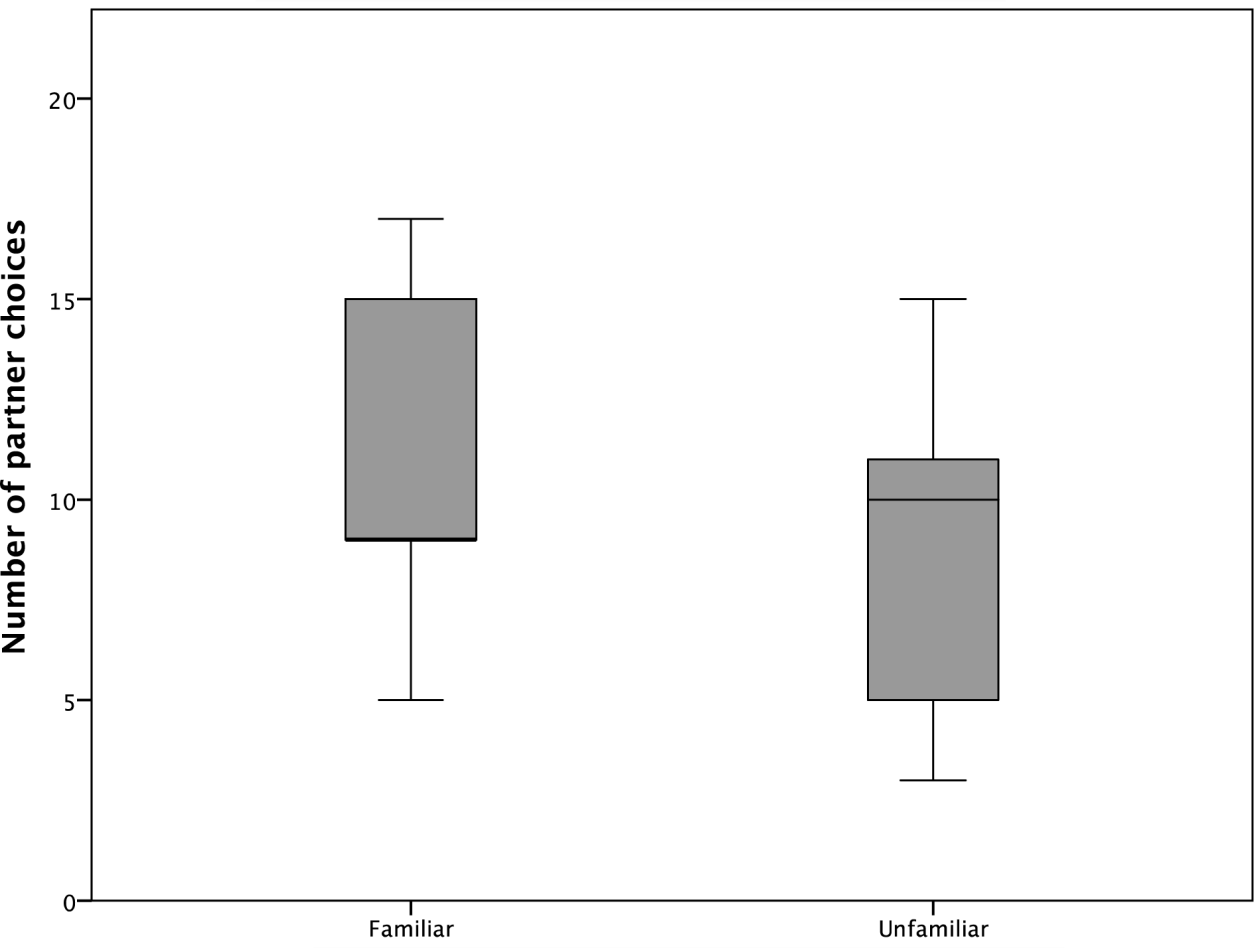
Median, inter-quartile range and range of the number of test subjects’ first choices for familiar and unfamiliar partners.

In experiment No. 1, subjects did not show a preference to cooperate with a specific partner based on familiarity; i.e., there was no significant difference between the amount of choices (out of 20) to cooperate with a familiar or unfamiliar partner (Wilcoxon: T^+^ = 26.5, n = 9, p = 0.631; Fig 4). 76,4% of these first choices ended with successful cooperation between the subject and that specific partner. Accordingly, comparisons of the number of successful trials show a similar pattern as first choices; i.e. there was no significant difference in successful trials with either familiar or unfamiliar partners (Wilcoxon: T^+^ = 30.5, N = 9, p = 0.341). Moreover, there was also no significant difference in success rates (# choices / # success) between cooperation with familiar and unfamiliar birds (Wilcoxon: T^+^ = 32.5, N = 9, p = 0.236).

**Fig 5:**
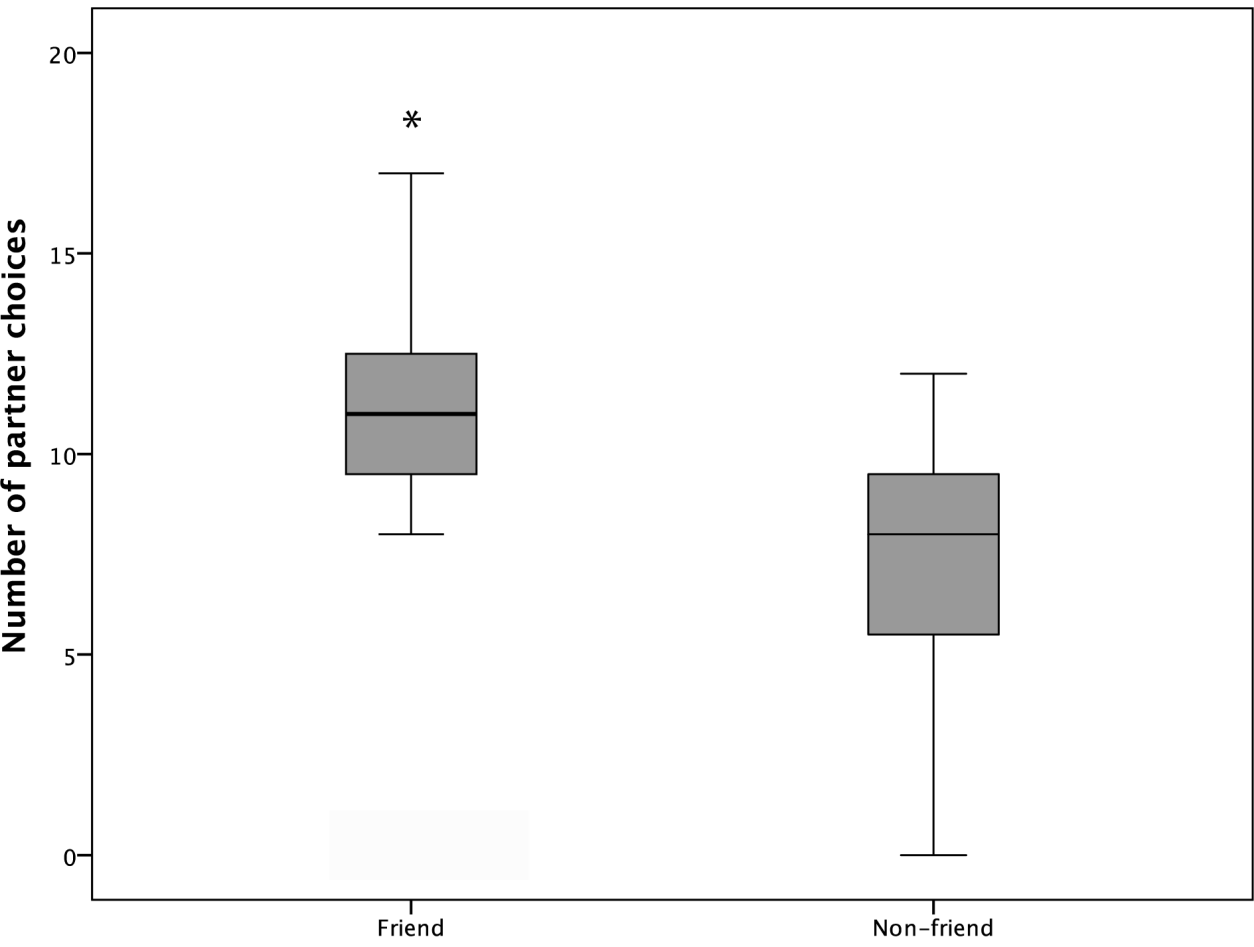
Median, inter-quartile range and range of the number of test subjects’ first choices for friends and non-friends; *p ≤ 0.05.

In contrast, subjects did have a significant preference to cooperate with their friends in experiment No. 2; i.e., they chose significantly more often to cooperate with their friend than with the non-friends (Wilcoxon: T^+^ = 39.5, n = 11, p = 0.043; Fig 5). 87.8% of these first choices ended with successful cooperation between the subject and that specific partner. Accordingly, comparisons of the number of successful trials show a similar pattern as first choices; i.e. compared to a random distribution subjects had significantly more successful cooperation trials with friends (Wilcoxon: T^+^ = 42.5, N = 11, p = 0.018). Moreover, the birds were also more successful when cooperating with a friend in comparison to when they choose to cooperate with the non-friend; i.e. there was a significant difference in success rate between cooperation with a friend and with a non-friend Wilcoxon: T^+^ = 27, N = 11, p = 0.028).

After their first choice, if still within the timeframe of a trial (< 2 min.), the subject bird could also try and cooperate with the other bird; i.e. it’s second choice, but could only be successful if that bird did not already pull and unthreaded the string (see S1 Video). In experiment 1 the subject bird made a second choice 83 times, and although these second-choice partners did not immediately pull in 50 of those trials, they waited successfully only three times (3.61%). In experiment 2 subject birds made a second choice 108 times, and although these second-choice partners did not immediately pull in 85 of those trials, they waited successfully in only 15 trials (13.89%). By the end of experiment 2, all but one bird had waited at least once when being second choice, and for 7 of the birds this waiting paid off at least once; i.e. after waiting they successfully cooperated with the subject. To study whether the birds may have learned this behavior throughout the first two experiments we ran binomial generalized linear mixed models (GLMM) taking into account how much experience partner birds had; i.e., number of trials as partner bird in a session and in total (over multiple sessions). The best fitting model on whether partner birds that were second choice waited or not only included the total trial numbers this individual already had as a partner as a significant factor (GLMM: F_1,420_ = 7.72, β = 0.018, p = 0.006); i.e. birds were more likely to wait when they had more experience as a partner. Moreover, this waiting also seemed to become more proficient when the birds had more experience, since the best fitting model on waiting success also only included the total trial numbers this individual already had as a partner as a factor, albeit not significantly (GLMM: F_1,133_ = 3.443, β = 0.020, p = 0.066). This suggests a learning effect where these partners learn to inhibit pulling immediately and start waiting for the subjects.

**Fig 6:**
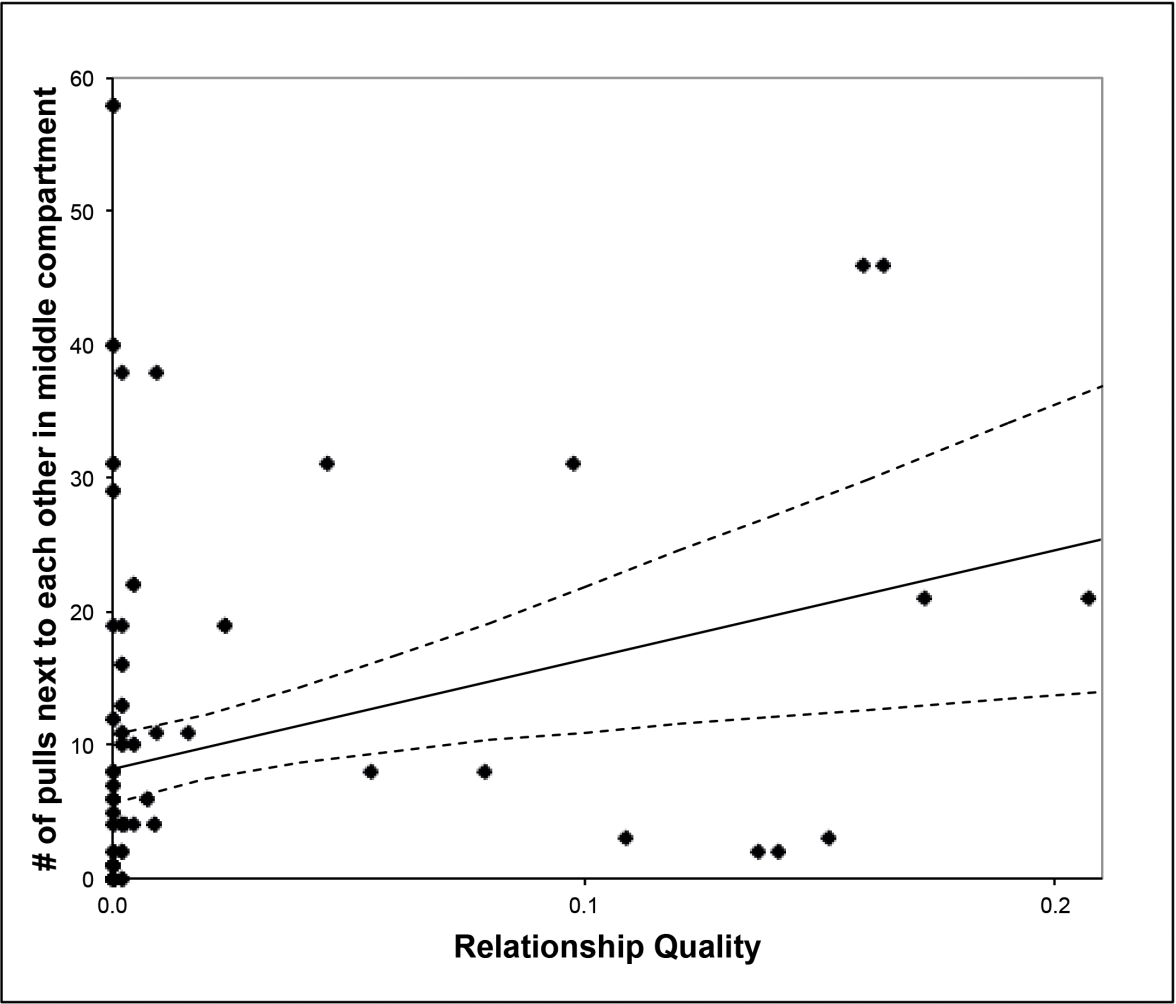
Relation between how often two subjects were standing next to each other in the center compartment (while cooperating with another bird) and their relationship quality.

In experiment 3, we did not find a significant correlation between relationship quality and how often two individuals cooperated with each other at either one of the apparatuses while controlling for rank distances (tau_rw;XY.Z_ = 0.047, n = 11, p = 0.336). In contrast, we did find a significant positive correlation between relationship quality and how often two individuals pulled side by side in the center compartment (tau_rw;XY.Z_ = 0.349, n = 11, p = 0.0037, Fig 6). Hence, independent of dominance ranks, the better the relationship quality between two individuals, the more often they both pulled standing next to each other in the center compartment, although they cooperated with other partners at different apparatuses (Fig 3).

## 4. Discussion

For this study we modified the loose string paradigm [37] and used two apparatuses in order to give ravens the choice between two partners to cooperate with. In contrast to most previous studies, this design allows for real-time cooperation coupled with partner choice and therefore closer resembles natural conditions (cf. [25,26]).

The ravens did not show a preference for either familiar or unfamiliar cooperation partners, nor did they cooperate more- or were more proficient in cooperating with either familiar or unfamiliar partners. This finding may reflect the high degree of fission-fusion dynamics in their social system [44]; i.e., individuals fuse regularly into groups of individuals with different degrees of familiarity. In such a social system it may be beneficial to not show any a priori preferences and quickly assess a newcomers’ willingness to cooperate [45].

The ravens did prefer to cooperate with their friends over their non-friends; i.e., they chose more often for friends, and subsequently, also cooperated more with friends. This supports observations of naturally occurring cooperation in wild and captive ravens [46]; it also supports earlier findings from dyadic experiments in the same group of ravens [12], and in other species [19–23]. Taken together, these findings suggest that animals show partner choice for cooperation. Moreover, cooperation among friends was also more proficient than cooperation among non-friends, suggesting that friends are better able to coordinate their actions in the loose-string paradigm. Again, this corroborates earlier findings from dyadic experiments in the same group of ravens [12], and in other species [19–23]. In this experiment it remains difficult, however, to deduce what is the proximate mechanism behind the choices made and the resulting proficiency. Do the ravens really choose to cooperate with friends (i.e. actual partner choice) and are they better able to coordinate their actions with friends, or are the ‘choices’ and increased efficiency due to less anxiety when approaching a friend rather than a non-friend (i.e. tolerance for proximity)? Therefore, we designed a third experiment where close proximity did not necessary lead to cooperation, and cooperation did not require the closest proximity.

In this open-choice experiment friends prioritized staying close over actually cooperating with them, either due to an active seeking of friends´ proximity or to avoiding non-friends that may ‘steal’ their rewards (cf. [12]). The results indicate that the preference to cooperate with friends may not be driven by an actual partner choice based on relationship quality as a whole, but rather due to spatial closeness resulting from a higher tolerance of proximity to close social associates, or from avoiding proximity to not so close associates. In accordance with our findings, another study that allowed partner choice in captive chimpanzees found no effect of affiliation on cooperation, but did find effects of kinship and rank distance [25]. In contrast to our results, a study in wild Barbary macaques showed that a strong social bond positively affected the maintenance of cooperation [26]. It should be noted, however, that in both studies there was only one apparatus and choices were thus limited; i.e. do I want to cooperate with the (high-ranking) animal already present, yes or no? Therefore, we advocate the use of multiple possibilities to cooperate in order to allow animals to make a free choice with whom to cooperate or not. Regarding ecological relevance, we, moreover, advocate the use of apparatuses that do not necessarily require close proximity (cf. cooperative hunting [28–31]).

A constrain of our last experiment is that there was wire-mesh between two possible cooperation partners, which differs from most previous studies on other animals (e.g. [19–23, 25, 26, 47, 48]; but see [13, 49, 50] that also have (mesh-wire) separations between individuals), making comparisons difficult. Furthermore, we cannot conclude what the effect of relationship quality and/or social tolerance would be on ravens cooperating in a completely open environment. Whereas in such an open environment the ravens may ‘actively’ choose not to cooperate with non-friends because these could displace them or steal food from them after cooperation [12], in our situation the ravens might not have needed to pay attention to their relationship to cooperation partners because of the physical separation. Nevertheless, all our ravens were able to move freely between the compartments and apparatuses; therefore, we can show that, when given the choice, they prefer to be in close proximity with their friends rather than to cooperate with them.

Although the preferences described do not necessarily require a full understanding of the paradigm, it is interesting to consider the cognitive mechanism at play and whether the ravens understood the role of the partner [51]. Previous experiments on these ravens suggested that they did not understand the need for the partner while cooperating, as they failed to inhibit pulling on one end of the loose-string in trials they either were alone or when they had to wait for a partner to come [12]. The current study had a similar condition hidden in its design, since the first two experiments were open choice experiments; i.e., after their first choice subjects could still go over to the other bird and also cooperate with that bird, given that this bird did not already pull the string and unthreaded it. If these partner birds would understand the task and/or would be able to inhibit pulling, they could also solve it when being second choice. Although at first this did not happen, during the first two experiments the birds started to wait and at the end of the first 2 experiments 7 of our 11 birds were successful while doing so at least once. Moreover, they waited more and became more proficient in waiting after experiencing more trials as partner, suggesting that they learned this during the experiments. This result highlights the crucial effect of experience / training in this paradigm [52], which should be taken into account when making comparisons among animal species. It remains unclear, however, what exactly the ravens learned; i.e., do they really understand the need for a partner, or do they, for example, learn to wait until they feel tension in the string. Therefore, future experiments should elucidate the exact cognitive mechanism, and see whether with enough experience ravens are at par with other species that pass this paradigm (e.g., apes [20, 21, 25, 47]; capuchin monkeys [49]; cotton-top tamarins [49]; hyenas [23]; dogs [53]; Asian elephants [50]; and coral trout [54]).

In conclusion, we showed that ravens can learn to wait for a partner in the loose-string paradigm. Moreover, we showed that ravens show marked preferences with whom they want to cooperate, but that these preferences, at least in our set-up, were motivated by tolerance for spatial proximity rather than actual partner choice based on relationship quality as a whole. Subsequently, our findings raise the important question if preferences in partner choice, found in non-open-choice cooperation studies, might not also originate from different levels of tolerance for proximity, rather than actual partner choice based on relationship quality as a whole. Note that to the observer the outcome is the same: animals cooperate with their friends rather than with non-friends. However, the proximate mechanism is different. Consequently, we advise to investigate the underlying, proximate mechanisms of partner choice in other species and urge the need for proximity controls in future animal cooperation studies.

## Supporting Information

S1 Video Showing a trial from experiment 2 in which the focal bird (Laggie) first chooses to cooperate with its friend; i.e., the bird on its right side (Adele), and then also cooperates with the other bird (Louise) which waited and did not yet pull. The left and right part of the video, show exactly the same trial, but shot from two different angles.
(MP4)
